# The effect of methylene blue infiltrating injection on anal pain after Milligan–Morgan surgery: A randomized controlled clinical study

**DOI:** 10.1097/MD.0000000000047613

**Published:** 2026-02-13

**Authors:** Jing Zhu, Heng Deng, Ming Li, Kun Tang

**Affiliations:** a Department of Anorectal Surgery, The South District of Hefei First People's Hospital, Hefei Binhu Hospital, Hefei, China; b Department of Proctology, The Second Hospital Affiliated Anhui University of Chinese Medicine, Hefei, China; c Department of Proctology, The First Affiliated Hospital of Anhui University of Chinese Medicine, Hefei, China.

**Keywords:** methylene blue, mixed hemorrhoids, pain management, randomized controlled trial

## Abstract

**Background:**

To investigate the efficacy and safety of methylene blue infiltrating injection (MBI) in alleviating postoperative pain following hemorrhoidectomy.

**Methods:**

In a randomized clinical trial, 60 patients with mixed hemorrhoids undergoing Milligan–Morgan surgery were divided into 2 groups: a study group (n = 30) and a control group (n = 30). Upon surgical completion, patients in the study group received an intraoperative MBI to the surgical incisions. All patients, in both groups, then received the same standard postoperative on-demand intravenous lornoxicam protocol. The primary outcome was anal pain intensity assessed by the visual analog scale scores at 6, 24, 48, and 72 hours. Secondary outcomes included limb movement score, incision edema, duration of the first postoperative defecation, supplemental analgesic consumption, length of hospital stay, hospitalization costs, and serum levels of substance P, 5-hydroxytryptamine, and prostaglandin E2.

**Results:**

No significant differences were observed in baseline characteristics. The study group exhibited significantly lower visual analog scale scores at all time points (all *P* < .001) and required substantially less supplemental lornoxicam (22.67 ± 17.01 mg vs 56.80 ± 9.32 mg, *P* < .001). Patients in the study group also had better limb movement scores, less incision edema, a shorter duration of the first postoperative defecation, a reduced hospital stay, and lower medical costs (*P* < .01). Serum levels of substance P, 5-hydroxytryptamine, and prostaglandin E2 were significantly lower in the study group (all *P* < .05). No perianal cellulitis, skin necrosis, or thrombosis occurred in either group.

**Conclusion:**

MBI provides effective and safe analgesia after Milligan–Morgan surgery, significantly reducing pain, analgesic consumption, hospital stay, and cost.

## 1. Introduction

Mixed hemorrhoids represent a prevalent anorectal condition, characterized by tissue congestion, edema, and the presence of blood in the stool.^[1]^ With the enhancement of living standards, the incidence of hemorrhoids has gradually increased in our country due to occupational, dietary, and other lifestyle factors,^[2]^ with a reported prevalence rate among our residents as high as 50.28%. Initially, conservative treatment approaches are typically employed to alleviate symptoms through lifestyle modifications, a high-fiber diet, adequate rest, and sclerotherapy.^[3]^ Cases where conservative treatments fail usually require surgical intervention, with the Milligan–Morgan procedure remaining a classic surgical method.^[4]^ While this operation addresses the clinical symptoms, postoperative issues such as pain, edema, and bleeding pose clinical challenges, particularly the intense postoperative pain that greatly hinders patient recovery.^[5]^ Given the variability in pain sensitivity among patients, the ability to standardize and simplify analgesic methods has been a quest for clinicians.^[6]^

A variety of analgesic strategies are available following mixed hemorrhoids surgery, including pharmacological interventions,^[7]^ acupuncture, moxibustion, and electroacupuncture. Medications such as opioid analgesics and nonsteroidal anti-inflammatory drugs are commonly used but can be associated with side effects including nausea, vomiting, and gastrointestinal bleeding, with long-term use potentially leading to addiction.^[8]^ Complementary therapies require skilled administration and regular treatment sessions.

Methylene blue (MB), a cationic thiazine dye extensively utilized as a biological stain and chemical indicator, has been increasingly recognized for its potential analgesic properties.^[9]^ In the present study, methylene blue infiltrating injection (MBI) was administered to treat anal pain resulting from Milligan–Morgan surgery, with the aim of assessing its analgesic efficacy and safety profile.

## 2. Materials and methods

### 2.1. Study design

**Figure 1.**
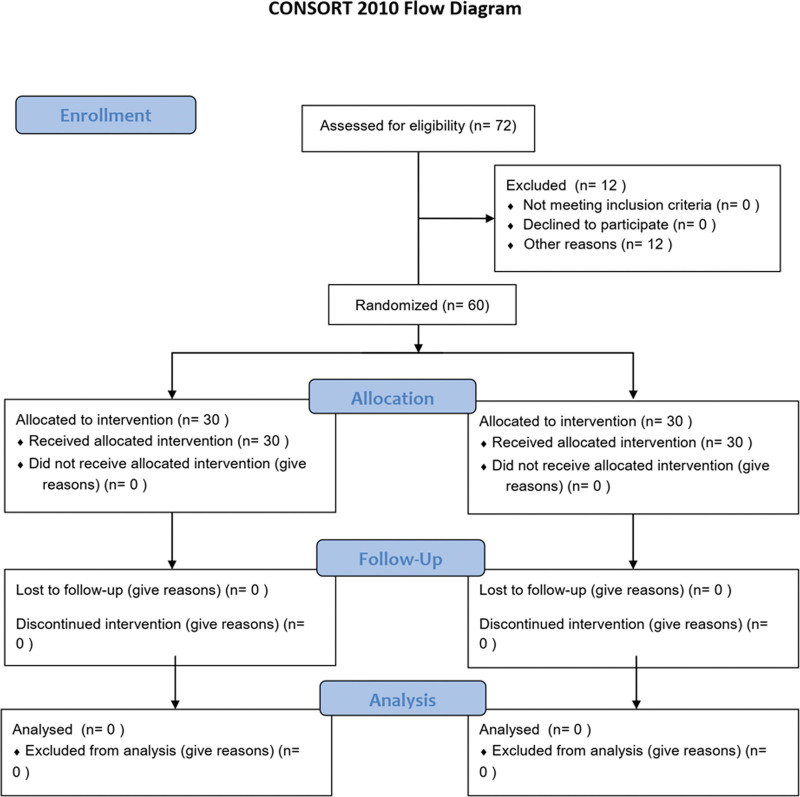
CONSORT flow diagram. All 60 randomized patients completed the study protocol and were included in the final analysis (no dropouts or loss to follow-up). CONSORT = Consolidated Standards of Reporting Trials.

A randomized controlled trial (ClinicalTrials.gov Identifier: NCT06660680) was meticulously designed and received ethical clearance from the Ethics Committee of Hefei Binhu Hospital (Ethical Approval Code: HFBH2021011). The study adhered to the 25-item checklist provided by the Consolidated Standards of Reporting Trials (CONSORT) statement.^[10]^ Commencing on November 1, 2024, and concluding on January 1, 2025, a cohort of 60 patients diagnosed with mixed hemorrhoids who underwent Milligan–Morgan surgery at our institution were recruited and randomly assigned in a 1:1 ratio to either the study group or the control group. The random allocation sequence was generated by an independent statistician using a computer-generated random number list. Allocation concealment was ensured through the use of sequentially numbered, opaque, sealed envelopes, which were opened only after the patient had provided written informed consent and just prior to surgery (Fig. 1). All 60 randomized patients completed the study protocol and were included in the final analysis (no dropouts or loss to follow-up). The criteria for participation: alignment with the diagnostic criteria for mixed hemorrhoids as outlined in the “American Society of Colon and Rectal Surgeons Clinical Practice Guidelines for the Management of Hemorrhoids”^[11]^; provision of informed consent following a thorough comprehension of the study’s aims; an age bracket of 18 to 75 years; satisfaction of surgical indications. Exclusion criteria encompassed: presence of anal fistula, anal fissure, perianal abscess, enteritis, or other intestinal pathologies; patients with known allergies; impaired liver or kidney function; andpregnancy or lactation.

### 2.2. Sample size estimation

The sample size was calculated a priori using GPower software (version 3.1.9.7, Düsseldorf, Germany). Based on the results of a pilot study and previous literature,^[12]^ the anticipated effect size (Cohen’s *d*) for the primary outcome (visual analog scale [VAS] score at 24 hours postoperatively) was set at 0.8. With a 2-sided significance level (α) of 0.05 and a desired statistical power (1 − β) of 0.80, the minimum required sample size was calculated to be 26 participants per group. To account for a potential dropout rate of approximately 10%, we planned to enroll 30 patients in each group, resulting in a total sample size of 60.

### 2.3. MBI procedure

**Figure 2.**
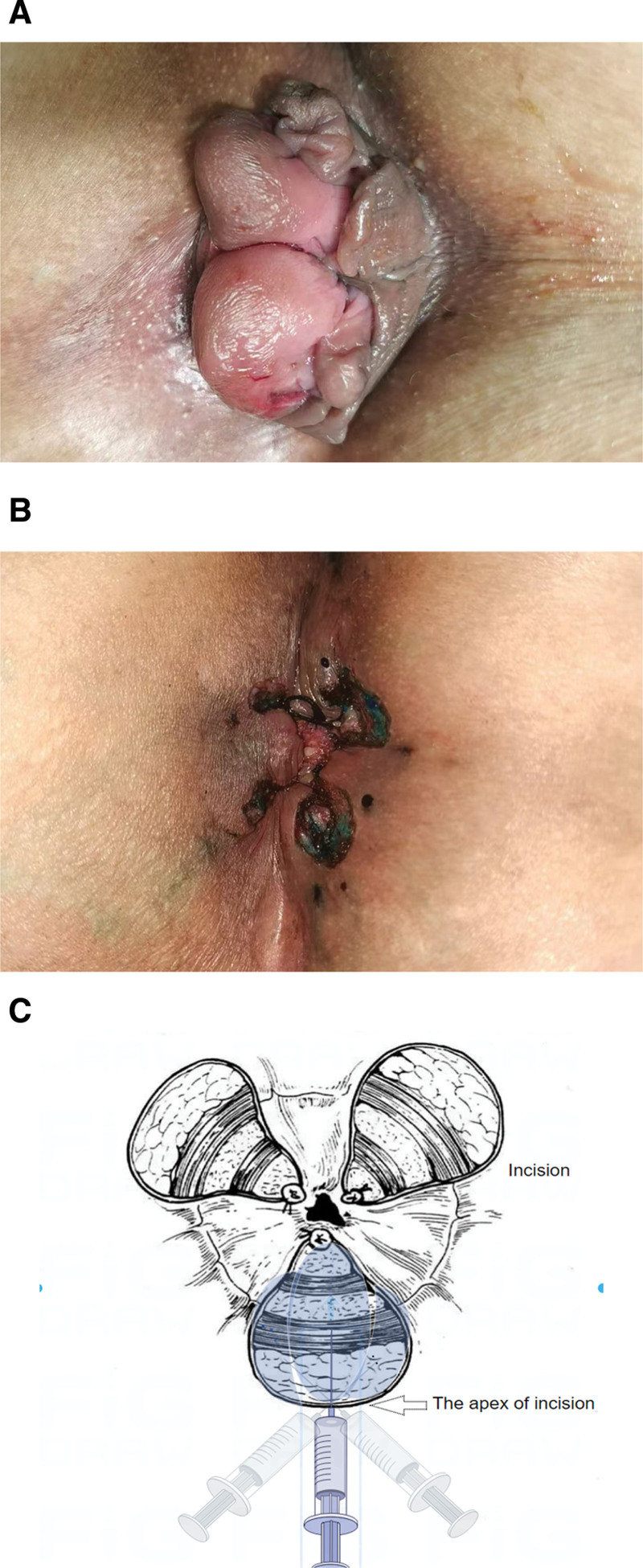
Intraoperative photographs and schematic of the methylene blue infiltrating injection (MBI) procedure. (A) A patient presenting with mixed hemorrhoids; (B) surgical incision following treatment with methylene blue infiltrating injection (MBI); (C) operation diagram of MBI therapy. BMI = blue infiltrating injection.

All patients underwent the Milligan–Morgan procedure under lumbar anesthesia while in the lithotomy position. The MB solution was prepared by blending 1 milliliter of a 1% methylene blue injection (manufactured by Jichuan Pharmaceutical Group Co., LTD., Taizhou, China, with the Sinomanical code H32024827; specification: 2 mL containing 20 mg) with 9 milliliters of normal saline. Upon completion of the hemorrhoidectomy, via subcutaneous infiltration, approximately 0.5 to 1 mL of the diluted methylene blue solution (0.1%) was injected per incision site in a fan-shaped distribution from the apex, directed evenly to the left, middle, and right, ensuring uniform blue staining of all incisional tissues. Subsequent to the injection, the incision was gently massaged for approximately 30 seconds to facilitate the dispersion. (Fig. 2) The total volume of solution injected per patient did not exceed 5 mL. All surgical procedures and MBI injections were performed by the same experienced colorectal surgeon (the first author) to minimize inter-operator variability.

### 2.4. Postoperative analgesic protocol

All patients, in both the study and control groups, received a standardized postoperative analgesic regimen. This consisted of on-demand intravenous lornoxicam (8 mg per bolus, with a minimum lockout interval of 6 hours), administered by nursing staff upon patient request for breakthrough pain. The MBI intervention was therefore an adjunctive, single intraoperative analgesic strategy applied in addition to this standard postoperative protocol. The efficacy of MBI was assessed by comparing the total consumption of this on-demand lornoxicam between groups, under the premise of an identical postoperative analgesic background.

### 2.5. Outcome measures

Primary and secondary outcomes:the primary outcome was the intensity of postoperative resting anal pain, assessed serially using the VAS at 6, 24, 48, and 72 hours after surgery. Secondary outcomes included: the limb movement score at 48 hours; incision edema score; duration of the first postoperative defecation; total consumption of on-demand intravenous lornoxicam; length of hospital stay; total medical cost; and serum levels of substance P (SP), 5-hydroxytryptamine (5-HT), and prostaglandin E2 (PGE2) at 48 hours postoperatively.

#### 2.5.1. Clinical assessments

Pain assessment: anal pain at rest was evaluated at 6, 24, 48, and 72 hours postoperatively using the visual analogue scale (VAS), ranging from 0 (no pain) to 10 (worst imaginable pain). Limb movement: assessed at 48 hours post-surgery using a 4-point scale: 0, unimpeded movement; 2, slow and stiff gait without support; 4, awkward gait requiring support; 6, inability to walk, confined to bed. Incisional edema: scored as: 0, no edema; 1, edema diameter ≤ 0.5 cm; 2, edema diameter 0.5 to 1 cm; 3, edema diameter ≥ 1 cm. The highest score was recorded. Other clinical parameters: the duration of the first postoperative defecation, total supplemental analgesic (lornoxicam) consumption, length of hospital stay, and total hospitalization cost were meticulously recorded and compared.

#### 2.5.2. Biochemical assessments (serum biomarkers):

To explore potential mechanisms, serum levels of pain-related mediators were quantified. At 48 hours post-surgery, a 3 mL fasting venous blood sample was drawn from each patient. Serum was separated by centrifugation at 2500 rpm for 20 minutes. Concentrations of SP, 5-HT, and PGE2 were measured using commercially available enzyme-linked immunosorbent assay (ELISA) kits (Amresco, Radnor) according to the manufacturer’s instructions. Absorbance was read at 450 nm, and concentrations were calculated based on standard curves.

### 2.6. Statistical analysis

Statistical analyses were performed using SPSS version 25 (IBM Corp., Chicago), and results were expressed as the mean ± standard deviation. For intergroup comparisons, either an independent samples *t*-test or the Mann–Whitney *U* test was applied, with the choice of statistical method being contingent upon the data distribution. Statistical significance was set at *P* < .05. Moreover, adjustments were made for potential confounding variables to guarantee the precision of the findings. In addition to the primary unadjusted analyses, a sensitivity analysis was preplanned to assess the robustness of the findings. Given a numerically higher mean number of hemorrhoids in the control group at baseline, an analysis of covariance (ANCOVA) was performed for all continuous outcomes, adjusting for the number of hemorrhoids as a covariate.

## 3. Results

### 3.1. Comparison of baseline characteristics between the study and control groups

At the baseline assessment, the control group exhibited a mean patient age of 47.428 ± 13.221 years, with 14 males among the cohort. The duration of mixed hemorrhoids was found to be 61.66 ± 9.693 months, and the average number of hemorrhoids was 4.7667 ± 1.5013. In contrast, the study group presented with a mean age of 50.59 ± 12.843 years, including 15 male patients. The duration of their condition was 62.08 ± 9.092 months, with a reduced number of hemorrhoids at 4.1034 ± 1.6112. No significant disparities were detected between the 2 groups with respect to age (95% confidence interval: −1.2 to 8, *P* = .2355), gender (95% CI: −2.9419e−05 to 2.1283e−05, *P* = .9093), duration of the disease (95% CI: −11 to 2.3, *P* = .2365), or the number of hemorrhoids (95% CI: −1.2368e−05 to 2, *P* = .0881). All baseline characteristics were well-balanced between groups (Table 1). A supplementary sensitivity analysis adjusting for the number of hemorrhoids (the variable with the largest numerical difference) yielded results fully consistent with the primary unadjusted analyses reported below.

**Table 1:** Baseline demographic and clinical characteristics of the study participants.

Characteristic	Study group (n = 30)	Control group (n = 30)	*P* value
Demographics			
Age, yr	50.6 ± 12.8	47.4 ± 13.2	.24
Sex, male	15 (50.0%)	14 (46.7%)	.80
Clinical features			
Number of hemorrhoids	4.10 ± 1.61	4.77 ± 1.50	.09
Disease duration, mo	62.1 ± 9.1	61.7 ± 9.7	.24

Data are presented as mean ± standard deviation or number (percentage).

*P* values were derived from independent samples *t*-test (continuous variables) or chi-square test (categorical variables).

MBI = methylene blue infiltrating injection.

### 3.2. Comparative analysis of therapeutic effects between the 2 groups

**Figure 3.**
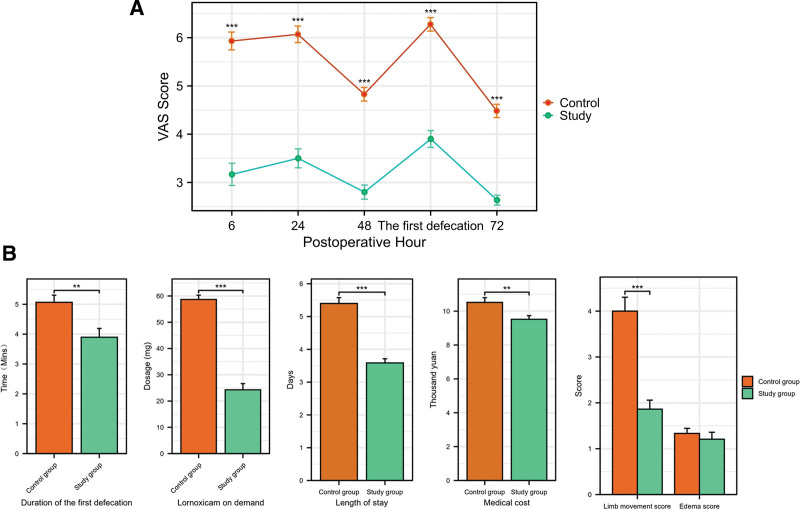
Comparison of clinical outcomes between the methylene blue infiltrating injection (MBI) group and the control group. (A) Comparison of VAS score between the 2 groups. VAS scores in the Study group were consistently and significantly lower than those in the control group. Data are presented as mean ± standard deviation. Error bars represent SD. Between-group comparisons were performed using independent samples *t*-test. ***, *P* < .001; **, *P* < .01. (B) Comparative analysis of limb movement score, incision edema score, duration of the first postoperative defecation, additional painkillers, length of stay and medical cost between the 2 groups. Data are presented as mean ± standard deviation. Error bars represent SD. Between-group comparisons were performed using independent samples *t*-test. ***, *P* < .001; **, *P* < .01. BMI = blue infiltrating injection, SD = standard deviation, VAS = visual analog scale.

The study group demonstrated significantly lower resting pain VAS scores at all postoperative time points (6, 24, 48, and 72 hours) compared to the control group (all *P* < .001; Fig. 3A). At 48 hours post-surgery, the mean limb movement score in the study group (1.87 ± 1.28) was markedly lower than that in the control group (4.13 ± 1.68), indicating better mobility and less pain-induced movement restriction (*P* < .001). The severity of incision edema was significantly reduced in the study group (mean score: 1.27 ± 0.91) compared to the control group (mean score: 1.67 ± 0.88; *P* < .05). The median duration of the first postoperative defecation was shorter in the study group (4.0 [IQR: 2.0–6.0] minutes) than in the control group (6.0 [IQR: 5.0–6.0] minutes; *P* < .01). The total postoperative consumption of on-demand intravenous lornoxicam was drastically lower in the study group (mean: 22.67 ± 17.01 mg) compared to the control group (mean: 56.80 ± 9.32 mg; *P* < .001). Consequently, the mean length of hospital stay was significantly shorter for patients receiving MBI (3.80 ± 0.85 days) than for controls (5.43 ± 1.01 days; *P* < .001). The reduced hospital stay and lower analgesic use contributed to lower total hospitalization costs in the study group (mean: ¥9.48 ± 1.26 thousand) compared to the control group (mean: ¥10.39 ± 1.56 thousand; *P* < .01; Fig. 3B).

### 3.3. Comparison of SP, 5-HT, and PGE2 concentrations across the 2 groups

Serum concentrations of key pain-related mediators measured at 48 hours postoperatively are summarized in Table 2. The mean serum SP concentration in the Study group was 58.36 ± 7.43 pg·mL^−1^, which was significantly lower than the 62.52 ± 8.65 pg·mL^−1^ observed in the control group (mean difference: −4.16 pg·mL^−1^, 95% CI: −7.42 to −0.90; *P* = .0135). Serum 5-HT levels were also markedly reduced in the study group (0.33 ± 0.10 pg·mL^−1^) relative to the control group (0.42 ± 0.12 pg·mL^−1^; mean difference: −0.09 pg·mL^−1^, 95% CI: −0.15 to −0.03; *P* = .0093). Similarly, the concentration of PGE2 was significantly lower in the study group (67.36 ± 14.62 pg·mL^−1^) than in the control group (82.33 ± 14.15 pg·mL^−1^; mean difference: −14.97 pg·mL^−1^, 95% CI: −25.04 to −4.90; *P* = .0365).

**Table 2:** Comparison of serum pain-related mediator concentrations between group.

Variables	Study groups	Control groups	Mean difference (95% CI)	*P* value
SP (pg·mL^-1^)	58.36 ± 7.43	62.52 ± 8.65	−4.16 (−7.42, −0.90)	.0135
5-HT (pg·mL^-1^)	0.33 ± 0.10	0.42 ± 0.12	−0.09 (−0.15, −0.03)	.0093
PGE 2 (pg·mL^-1^)	67.36 ± 14.62	82.33 ± 14.15	−14.97 (−25.04, −4.90)	.0365

Data are presented as mean ± standard deviation. Between-group comparisons were performed using independent samples *t*-test. The mean difference (95% confidence interval) is presented for the study group relative to the control group.

5-HT = 5-hydroxytryptamine, SP = substance P.

## 4. Discussion

In this study, a randomized controlled clinical trial was undertaken to investigate the impact of MBI on post-Milligan–Morgan surgery anal pain. The findings revealed that MBI significantly alleviated clinical parameters including VAS scores, limb movement scores, and the required dosage of intravenous lornoxicam. Beyond the clinical metrics, our study provides mechanistic insights by quantifying key pain-related mediators. The significant reduction in serum levels of SP, 5-HT, and PGE2 in the study group offers biochemical corroboration for the observed clinical analgesia. These mediators are established key players in the pathophysiology of postoperative pain: SP and 5-HT are pivotal in driving neurogenic inflammation and peripheral sensitization,^[13]^ and their elevated levels have been directly correlated with heightened pain intensity in various surgical contexts.^[14]^ Similarly, PGE2 is a well-known promoter of hyperalgesia.^[15]^ The attenuation of these mediators by MBI aligns with its documented anti-inflammatory and neuromodulatory properties, suggesting that its analgesic effect is mediated, at least in part, through the suppression of this pro-inflammatory and pro-nociceptive signaling cascade. Furthermore, MBI was found to abbreviated the duration of hospital stay, thereby contributing to reduced medical expenses. Notably, no adverse events such as perianal cellulitis, skin necrosis, or perianal thrombosis were encountered in either group throughout the trial.

Postoperative analgesia for hemorrhoidal procedures is crucial in anorectal surgery.^[16]^ Postoperative pain not only impedes patient recovery and diminishes quality of life but may also result in complications such as urinary retention,^[17]^ constipation, and perianal edema.^[18]^ Currently, clinical analgesia following hemorrhoidectomy faces several challenges. The complexity of postoperative hemorrhoidal pain mechanisms, involving a multitude of physiological and pathological factors, is a primary issue. Surgical incisions, suture ligation, and subsequent wound inflammation are all potential pain sources.^[19]^ Additionally, while commonly employed analgesics such as opioids and nonsteroidal anti-inflammatory drugs can mitigate pain, they are not without drawbacks, including the risks of addiction, medication delay,^[20]^ gastrointestinal side effects, and hepatorenal damage.^[21]^ Although patient-controlled analgesia and similar methods have their merits, their high costs and limited accessibility present barriers to widespread adoption.^[22]^

Methylene blue has detoxification, anti-inflammatory, and analgesic properties, and this clinically inexpensive drug has been shown to be effective in reducing postoperative pain. It effectively curbs the activation of STAT3 and intercepts the surge in interleukin-6 levels.^[23]^ The analgesic mechanism of methylene blue is primarily linked to its anti-inflammatory action, reduction in sodium currents,^[24]^ and reversible denervation processes.^[25]^ Methylene blue exhibits strong neurophilic properties. It induces a reversible demyelinating effect on the peripheral nerve medulla, thereby blocking pain signal conduction and producing a definitive and long-lasting analgesic effect.

It is noteworthy that the regeneration of the nerve medulla typically occurs within approximately 30 days, a period that coincides with the substantial healing of hemorrhoidal incisions. In the domain of postoperative hemorrhoidal analgesia, the administration of methylene blue aligns with the principles of preventive analgesia, avoids medication delay. Subcutaneous injection of a 0.1% methylene blue solution has proven to be an effective measure for mitigating pain following anal canal surgeries, albeit primarily supported by retrospective studies,^[26]^ or case report,^[27]^ or lack of objective evidence for pain indicators.^[28]^

This study has several limitations. First, due to the distinctive blue discoloration of tissues and urine associated with methylene blue, effective blinding of participants and care providers was not feasible. This introduces a risk of performance and detection bias, particularly for subjective outcomes like VAS scores. However, we sought to mitigate this by incorporating objective measures such as serum biomarker levels and analgesic consumption. Second, the follow-up period was limited to 72 hours postoperatively, which captures the acute pain phase but not long-term recovery or recurrence. Third, while our sample size was adequate based on a priori calculation, a larger multicenter trial would enhance the generalizability of our findings. Lastly, the control group received analgesia only on-demand, whereas the intervention was a single intraoperative administration. This difference in analgesic strategy, though reflective of certain clinical practices, may have contributed to the observed effect size. Future studies could employ an active comparator with standardized postoperative analgesia in both groups.

## 5. Conclusion

The MBI has demonstrated a marked analgesic effect on post-Milligan–Morgan surgery anal pain, while also exhibiting an impressive safety profile.

## Acknowledgments

We thank all participants and nursing staff for their cooperation. We also confirm that all individuals named in this Acknowledgments section have provided permission to be listed. H.D. and M. L. are contributed equally to this work.

## Author contributions

**Conceptualization:** Heng Deng.

**Formal analysis:** Kun Tang.

**Funding acquisition:** Ming Li.

**Investigation:** Kun Tang.

**Methodology:** Ming Li, Kun Tang.

**Resources:** Ming Li.

**Software:** Jing Zhu.

**Supervision:** Heng Deng.

**Visualization:** Ming Li.

**Writing − original draft:** Jing Zhu.
